# Supplementary material for: Risk factors for diarrheagenic Escherichia coli infection in children aged 6–24 months in peri-urban community, Nairobi, Kenya
Source: PLOS Glob Public Health. 2023 Nov 22;3(11):e0002594. doi: 10.1371/journal.pgph.0002594 (PMC10664883; doi:10.1371/journal.pgph.0002594)
Supplement: S1 File — (DOCX) [file pgph.0002594.s001.docx]

**S1 File.** **PCR conditions for *Escherichia coli* pathotyping**

**PCR detection of *E. coli* O157 stx 1, stx2 and eae genes (WHO Global Sulm-surv, 2003)**:

Multiplex PCR for sxt1 and stx2

For each sample, a reaction volume of 25µl was used. This constituted 12.5µl of 2x PCR mastermix, 0.5µl each of the primers:

1. Stx1 forward primer 5’-CAGTTAATGTGGTGGCGAAGC-3’
2. Stx1reverse primer 5’-CACCAGACAATGTAACCGCTG-3’
3. Stx2 forward primer 5’-ATCCTATTCCCGGGAGTTTACG-3’
4. Stx2 reverse primer 5’-GCGTCATCGTATACACAGGAGC-3’

The volume was topped upto 22µl with nuclease-free water and a 3µl of DNA tempalate added. Thermal cycling conditions consisted of initial denaturation of 94^o^C for 5 minutes, followed by 35 cycles of denatration at 94^o^C for 1 minute, annealing at 57^o^C for 1 munite, extension at 72^o^C for 1 minute. A final extension was done at 72^o^C for 10 minutes and final hold at 4^o^C. Expected PCR product sizes were 348bp and 584bp for stx1 and 2 respectively.

Intimin gene (eae) was screened using the forward primer 5’-CCCGAATTCGGCACAAGCATAAGC -3’ and reverse primer 5’-CCCGGATCCGTCTCGCCAGTATTCG-3’ using the same thermal cycling conditions with the annealing temperature adjusted to 52^o^C. The expected PCR product size was 863bp.

**Multiplex PCR detection of EPEC (450bp, 229bp) and ETEC (322bp, 170bp)**:

The primers used for multiplex PCR detection of EPEC and ETEC:

1. Enterotoxigenic E. coli (ETEC)

i. heat labile gene (etl) forward 5’- CTCTATGTGCACACGGAGC-3’; reverse 5’- CCATACTGATTGCCGCAAT-3’

ii. heat stable gene (stlA) forward 5’- TCTTTCCCCTCTTTTAGTCAGTC-3’; reverse 5’- CCGCACAGGCAGGATTAC-3’

1. Enteropathogenic E. coli (EPEC)

i. bundle forming pilus protein (bfpA) forward 5’- CACCGTTACCGCAGGTGTGA-3’; reverse 5’- GTTGCCGCTTCAGCAGGAGT-3’

ii. intimin gene (eaeA) forward 5’- TGATAAGCTGCAGTCGAATCC-3’; reverse 5’- CTGAACCAGATCGTAACGGC-3’ (Hegde 2012).

For each sample, a 25µl reaction volume was prepared. This was done by mixing 12.5µl of 2x PCR mastermix with 0.5µl of each of the eight primers. The mix was topped up to 22µl with nuclease-free water. Finally, 3µl of template DNA was added. The thermal cycling conditions consisted of an initial denaturation 95^o^C for 1 minute. This was followed by 35 cycles of 94^o^C denaturation for 1minute, annealing at 56^o^C for 1 minute, extension at 72^o^C for 1 minute. Final extension was at 72^o^C for 5 minutes followed by final hold at 4^o^C. Gel electrophoresis was doen on 2% agarose in 1x TAE buffer and staining using SYBR safe dye. The expected amplicon sizes were 322bp (elt), 170bp (stla), 450bp (bfpA) and 229bp (eaeA).

**Multiplex PCR detection of EIEC (933bp & 618bp), EAEC (1111bp & 378bp) and DAEC (542bp):** Cocktail mix was prepared by adding appropriate quantity of 2x PCR mastermix, nuclease-free water and 0.5µl of each of the following primers:

Enteroinvasive E. Coli (EIEC)

1. invasion plasmid antigen H gene (ipaH) forward primer 5’-CTCGGCACGTTTTAATAGTCTGG-3’, reverse rpimer 5’-GTGGAGAGCTGAAGTTTCTCTGC-3’;
2. transcriptional regulator virF forward primer 5’-AGCTCAGGCAATGAAACTTTGAC-3’, virF reverse primer 5’-TGGGCTTGATATTCCGATAAGTC-3’.

Enteroaggregative E. Coli (EAEC)

1. Serine protease autotransporter (pic) forward 5’-AGCCGTTTCCGCAGAAGCC-3’; reverse primer 5’-AAATGTCAGTGAACCGACGATTGG-3’;
2. aggregative adherence fimbria II (aafII) forward primer 5’-CACAGGCAACTGAAATAAGTCTGG-3’, aafII reverse primer 5’-ATTCCCATGATGTCAAGCACTTC-3’.

Diffusely Adherent E. Coli (DAEC)

1. Dr adhesine family protein (DaaE) forward 5’-GAACGTTGGTTAATGTGGGGTAA-3’, reverse 5’-TATTCACCGGTCGGTTATCAGT-3’ (Chandra et al. 2013)**.**

EIEC, EAEC and DAEC primers were multiplexed in a single PCR reaction. The cocktail mix was prepared as illustrated in table 1. A 3µl of DNA template was added to an aliquot of 15µl of cocktail mix. Thermal cycling conditions consisted of an initial denaturation of 94^o^C for 5minutes followed by 35 cylces of denaturation at 94^o^C for 30sec, annealing at 59^o^C for 30sec and extension at 72^o^C for 1 minute. Final extension was at 72^o^C for 5 minutes and a final hold at 4^o^C. The PCR products were resolved on a 1.5% agarose with 1x TAE buffer, expecting 933bp, 618bp, 1111bp, 378bp and 542 bp for ipaH, virF, pic, aafII and daaE genes respectively.

Table 1 Preparation of PCR cocktail mix

| **Reagent** | | **1 reaction (µl)** | **100 reactions (µl)** |
| --- | --- | --- | --- |
| PCR mastermix (2X) | | 9 | 900 |
| EIEC | ipaH F | 0.5 | 50 |
|  | ipaH R | 0.5 | 50 |
|  | virF F | 0.5 | 50 |
|  | virF R | 0.5 | 50 |
| EAEC | pic F | 0.5 | 50 |
|  | pic R | 0.5 | 50 |
|  | aafII F | 0.5 | 50 |
|  | aafII R | 0.5 | 50 |
| DAEC | daaE F | 0.5 | 50 |
|  | daaE R | 0.5 | 50 |
| Nuclease-free H_2_O | | 1 | 100 |
| DNA template | Aliquot 15µl of cocktail mix into PCR tubes and add 3µl of DNA template | | |

**Multiplex PCR detection of non-O157 STEC:**

Cocktail mix was prepared following the illustration in table 2 for a 20µl reaction volume. A 3µl DNA template was added to an aliquot of 17µl of the cocktail mix. Primers used were specific to the virulence factors used to characterise the different *E. coli* pathotypes.

These included

1. O26 (forward 5’-GGGGGTGGGTACTATATTGG-3’; reverse 5’- AGCGCCTATTTCAGCAAAGA-3’),
2. O45 (forward 5’- GGGCTGTCCAGACAGTTCAT-3’; reverse 5’- TGTACTGCACCAATGCACCT-3’),
3. O103 (forward 5’- TAAGTACGGGGGTGCTTTTT-3’; reverse 5’- AAGCTCCCGAGCACGTATAA-3’),
4. O111 (forward 5’- CAAGAGTGCTCTGGGCTTCT-3’; reverse 5’- AACGCAAGACAAGGCAAAAC-3’),
5. O121 (forward 5’- TCATTAGCGGTAGCGAAAGG-3’; reverse 5’- TTCTGCATCACCAGTCCAGA-3’),
6. O145 (forward 5’- TGCTCGACTTTTACCATCAAC-3’; reverse 5’- AACCAACACCATACACCTTGTCTT-5’) (Paddock et al. 2012).

Thermal cycling conditions consisted of an initial denaturation at 94^o^C for 5 minutes, followed by 35 cycles of denaturation at 94^o^C for 30sec, annealing at 55^o^C for 80sec, extension at 67^o^C for 80sec. Final extension was at 68^o^C for 7 minutes and final hold at 4^o^C.

Table 2 Preparation of PCR cocktail mix

| Reagent | | 1 reaction (µl) | 10 reactions (µl) |
| --- | --- | --- | --- |
| PCR mastermix (2X) | | 10 | 100 |
| O26 | F | 0.5 | 5 |
|  | R | 0.5 | 5 |
| O45 | F | 0.5 | 5 |
|  | R | 0.5 | 5 |
| O103 | F | 0.5 | 5 |
|  | R | 0.5 | 5 |
| O111 | F | 0.5 | 5 |
|  | R | 0.5 | 5 |
| O121 | F | 0.5 | 5 |
|  | R | 0.5 | 5 |
| O145 | F | 0.5 | 5 |
|  | R | 0.5 | 5 |
| Nuclease-free H_2_O | | 1 | 10 |
| DNA template | Aliquot 17µl of cocktail mix into PCR tubes and add 3µl of DNA template | | |
