# Supplementary material for: Risk factors for diarrheagenic Escherichia coli infection in children aged 6–24 months in peri-urban community, Nairobi, Kenya
Source: PLOS Glob Public Health. 2023 Nov 22;3(11):e0002594. doi: 10.1371/journal.pgph.0002594 (PMC10664883; doi:10.1371/journal.pgph.0002594)
Supplement: S1 Table — (DOCX) [file pgph.0002594.s003.docx]

S1 Table. Variables lowering Odds of DEC carriage in children

| **Predictor** | **Number of observations** | **Number positive (%)** | **OR (95%CI)** | **p-value** |
| --- | --- | --- | --- | --- |
| **Diarrhea past week** |  |  |  |  |
| No | 436 | 94 (21.6) | 1 | Reference |
| Yes | 104 | 19 (18.3) | 0.76 (0.75-0.76) | **<0.001** |
| **Food Type Biscuits** |  |  |  |  |
| No | 497 | 111 (22.3) | 1 | Reference |
| Yes | 43 | 2 (4.7) | 0.15 (0.15-0.16) | **<0.001** |
