# Supplementary material for: Risk factors for diarrheagenic Escherichia coli infection in children aged 6–24 months in peri-urban community, Nairobi, Kenya
Source: PLOS Glob Public Health. 2023 Nov 22;3(11):e0002594. doi: 10.1371/journal.pgph.0002594 (PMC10664883; doi:10.1371/journal.pgph.0002594)
Supplement: S2 Table — (DOCX) [file pgph.0002594.s004.docx]

S2 Table. Variables with no association with DEC carriage in children

| **Predictor** | **Number of observations** | **Number positive (%)** | **OR (95%CI)** | **p-value** |
| --- | --- | --- | --- | --- |
| **Neighbour own (chicken)** | |  |  |  |
| No | 280 | 53 (18.9) | 1 | Reference |
| Yes | 260 | 60 (23.1) | 1.33 (0.86-2.06) | 0.20 |
| **Neighbour own (cat)** | |  |  |  |
| No | 255 | 48 (18.8) | 1 | Reference |
| Yes | 285 | 65 (22.8) | 1.34 (0.86-2.10) | 0.20 |
| **Any rodents' sign** |  |  |  |  |
| No | 199 | 41 | 1 | Reference |
| Yes | 341 | 72 | 1.06 (0.67-1.67) | 0.799 |
| **No. of household members** | |  |  |  |
| 4 or less | 322 | 70 | 1 | Reference |
| over 4 | 218 | 43 | 0.85 (0.54-1.33) | 0.483 |
| **Animal faeces on premises** | |  |  |  |
| No | 504 | 106 | 1 | Reference |
| Yes | 36 | 7 | 0.87 (0.36-2.12) | 0.762 |
| **Garbage visible on premises** | |  |  |  |
| No | 357 | 79 | 1 | Reference |
| Yes | 183 | 34 | 0.78 (0.48-1.26) | 0.307 |
