# Supplementary material for: Risk factors for diarrheagenic Escherichia coli infection in children aged 6–24 months in peri-urban community, Nairobi, Kenya
Source: PLOS Glob Public Health. 2023 Nov 22;3(11):e0002594. doi: 10.1371/journal.pgph.0002594 (PMC10664883; doi:10.1371/journal.pgph.0002594)
Supplement: S3 Table — (DOCX) [file pgph.0002594.s005.docx]

**S3 Table. Comparison of mixed-effects logistic regression models for risk factors for diarrheagenic E. coli carriage in 6-24 months old children.**

| Model | Model formula | AIC |
| --- | --- | --- |
| 1 | pathogenic.e.coli ~ agegroup + surveyFood_Typematoke + surveyFood_Typepulses_legumes +  surveyFood_Typebroth + foodreheat1 + (1 \| CHV_ID) | 541.8 |
| 2 | pathogenic.e.coli ~ agegroup + surveyFood_Typematoke + surveyFood_Typepulses_legumes + foodreheat1 + (1 \| CHV_ID) | 541.0 |

For each model, the model formula with their Akaike Information Criterion (AIC) values are provided. CHV-ID – community health volunteer identity
