# Supplementary material for: Risk factors for diarrheagenic Escherichia coli infection in children aged 6–24 months in peri-urban community, Nairobi, Kenya
Source: PLOS Glob Public Health. 2023 Nov 22;3(11):e0002594. doi: 10.1371/journal.pgph.0002594 (PMC10664883; doi:10.1371/journal.pgph.0002594)
Supplement: Attachment — Submitted filename: PGPH-D-23-01426_revised.pdf [file pgph.0002594.s006.pdf]

PLOS Global Public Health

# Risk factors for diarrheagenic Escherichia coli infection in children aged 6-24 months in peri-urban community, Nairobi, Kenya

--Manuscript Draft--

| | |
|---|---|
| **Manuscript Number:** | PGPH-D-23-01426 |
| **Article Type:** | Research Article |
| **Full Title:** | Risk factors for diarrheagenic Escherichia coli infection in children aged 6-24 months in peri-urban community, Nairobi, Kenya |
| **Short Title:** | Risk factors for diarrheagenic Escherichia coli in children |
| **Corresponding Author:** | Noah Okoth Okumu<br>International Livestock Research Institute<br>Nairobi, Nairobi KENYA |
| **Order of Authors:** | Noah O. Okumu |
| | Joseph J. N. Ngeranwa |
| | Dishon M. Muloi |
| | Linnet Ochieng |
| | Arshnee Moodley |
| | Christine Mutisya |
| | Alice Kiarie |
| | Joseph O. Wasonga |
| | Julie Watson |
| | Maud Akissi Amon-Tanoh |
| | Oliver Cumming |
| | Elizabeth A. J. Cook |
| **Keywords:** | Diarrheagenic E. coli; Pathotypes; Children; Community; low-income settings |
| **Abstract:** | Escherichia coli commonly inhabits the gut of humans and animals as part of their microbiota. Though mostly innocuous, some strains have virulence markers that make them pathogenic. This paper presents results of a cross-sectional epidemiological study examining prevalence of diarrheagenic E. coli (DEC) pathotypes in stool samples of presumptive healthy children (n=540) in Dagoretti South subcounty, Nairobi, Kenya. E. coli was cultured and pathotyped using PCR to target specific virulence markers associated with Shiga-toxin, enteropathogenic, enterotoxigenic, enteroaggregative, enteroinvasive and diffusely adherent E. coli. Overall prevalence of DEC pathotypes was 20.9% (113/540) with enteropathogenic E. coli being the most prevalent (35.4%), followed by enteroaggregative E. coli (24.4%) and Shiga-toxin producing E. coli (22.8%) among positive samples. We found evidence of co-infection with multiple pathotypes in 11.5% of the positive samples. Our models indicated that at the household level, carriage of DEC pathotypes in children was associated with age group [12-18 months] (OR 1.78; 95%CI 1.03-3.07; p=0.04), eating matoke (OR 2.32; 95%CI 1.44-3.73; p=0.001) and pulses/legumes (OR 1.74; 95%CI 1.01-2.99; p=0.046) while livestock ownership or contact showed no significant association with DEC carriage (p>0.05). Our findings revealed significant prevalence of pathogenic DEC circulating among presumptive healthy children in the community. Since there has been no previous evidence of an association between any food type and DEC carriage, unhygienic handling and preparation of matoke and pulses/legumes could be the reason for significant association with DEC carriage. Children 12-18 months old are more prone to DEC infections due to exploration and hand-to-mouth behavior. A detailed understanding is required on what proportion of positive cases developed severe symptomatology as well as fatal outcomes. The co-infection of pathotypes in the rapidly urbanizing environment needs to be investigated for hybrid or hetero- |

Powered by Editorial Manager® and ProduXion Manager® from Aries Systems Corporation

| | |
|---|---|
| | pathotype circulation that have been implicated in previous infection outbreaks. |
| Additional Information: | |
| Question | Response |
| **Financial Disclosure**<br><br>Enter a financial disclosure statement that describes the sources of funding for the work included in this submission and the role the funder(s) played. This includes grants and any commercial funding of the work or authors.<br><br>This statement will be typeset if the manuscript is accepted for publication.<br><br>*Please review the submission guidelines and the instructions link below for detailed requirements and guidance.* | Funding statement<br>The project Development of a comprehensive intervention to address foodborne enteric disease risks among young children living in low-income informal neighbourhoods of Maputo and Nairobi is supported by the Bill & Melinda Gates Foundation (BMGF) and the Foreign, Commonwealth and Development Office (FCDO) of the UK Government (INV-008449) and the CGIAR Research Program on Agriculture for Nutrition and Health, which is led by the International Food Policy Research Institute (IFPRI). |
| **Competing Interests**<br><br>On behalf of all authors, disclose any competing interests that could be perceived to bias this work.<br><br>This statement will be typeset if the manuscript is accepted for publication.<br><br>*Please review the instructions link below and PLOS Global Public Health's competing interests policy to determine what information must be disclosed at submission.* | The authors declare that the research was conducted without any commercial or financial relationships that could be perceived as a potential conflict of interest. |
| **Data Availability**<br><br>Before publication, Authors are required to make fully available and without restriction all data underlying their findings. Please see our PLOS Data Policy page for detailed information on this policy. | Data is contained within the article. |

Powered by Editorial Manager® and ProduXion Manager® from Aries Systems Corporation

| | |
|---|---|
| A **Data Availability Statement**, detailing where the data can be accessed, is required at first submission. Insert your Data Availability Statement in the box below.<br><br>Please see the data reporting section of our submission guidelines for instructions on what you need to include in your Data Availability Statement.<br><br>This statement will be typeset if the manuscript is accepted for publication.<br><br>*PLOS allows rare exemptions to address legal and ethical concerns. If you have legal or ethical restrictions, please use the box below to detail these in full sentences for the Journal team to consider.* | |

Manuscript Click here to access/download;Manuscript;Manuscript.docx

# Risk factors for diarrheagenic *Escherichia coli* infection in children aged 6-24 months in peri-urban community, Nairobi, Kenya

Noah O. Okumu[1,2,*], Joseph J. N. Ngeranwa[2], Dishon M. Muloi[1,5], Linnet Ochieng[1], Arshnee Moodley[1,3], Christine Mutisya[1], Alice Kiarie[1], Joseph O. Wasonga[1], Julie Watson[4], Maud Akissi Amon-Tanoh[4], Oliver Cumming[4], Elizabeth A. J. Cook[1]

[1]Animal and Human Health Department, International Livestock Research Institute, Nairobi, Kenya

[2]Department of Biochemistry, Biotechnology and Microbiology, Kenyatta University, Kenya

[3]Department of Veterinary and Animal Sciences, University of Copenhagen, Frederiksberg C, Denmark

[4]Department of Disease Control, London School of Hygiene and Tropical Medicine, United Kingdom

[5]Institute of Infection, Veterinary and Ecological Sciences, University of Liverpool, Liverpool, United Kingdom

*Corresponding author

Email: N.Okumu@cgiar.org (NOO), E.cook@cgiar.org (EAJC)

## Abstract

*Escherichia coli* commonly inhabits the gut of humans and animals as part of their microbiota. Though mostly innocuous, some strains have virulence markers that make them pathogenic. This paper presents results of a cross-sectional epidemiological study examining prevalence of diarrheagenic *E. coli* (DEC) pathotypes in stool samples of presumptive healthy children (n=540) in Dagoretti South subcounty, Nairobi, Kenya. *E. coli* was cultured and pathotyped using PCR to target specific virulence markers associated with Shiga-toxin, enteropathogenic, enterotoxigenic, enteroaggregative, enteroinvasive and diffusely adherent *E. coli*. Overall prevalence of DEC pathotypes was 20.9% (113/540) with enteropathogenic *E. coli* being the most prevalent (35.4%), followed by

enteroaggregative *E. coli* (24.4%) and Shiga-toxin producing *E. coli* (22.8%) among positive samples. We found evidence of co-infection with multiple pathotypes in 11.5% of the positive samples. Our models indicated that at the household level, carriage of DEC pathotypes in children was associated with age group [12-18 months] (OR 1.78; 95%CI 1.03-3.07; p=0.04), eating matoke (OR 2.32; 95%CI 1.44-3.73; p=0.001) and pulses/legumes (OR 1.74; 95%CI 1.01-2.99; p=0.046) while livestock ownership or contact showed no significant association with DEC carriage (p>0.05). Our findings revealed significant prevalence of pathogenic DEC circulating among presumptive healthy children in the community. Since there has been no previous evidence of an association between any food type and DEC carriage, unhygienic handling and preparation of matoke and pulses/legumes could be the reason for significant association with DEC carriage. Children 12-18 months old are more prone to DEC infections due to exploration and hand-to-mouth behavior. A detailed understanding is required on what proportion of positive cases developed severe symptomatology as well as fatal outcomes. The co-infection of pathotypes in the rapidly urbanizing environment needs to be investigated for hybrid or hetero-pathotype circulation that have been implicated in previous infection outbreaks.

**Keywords:** Diarrheagenic *E. coli*, Pathotypes, Children, Community, low-income settings

# Introduction

*Escherichia coli* is the predominant commensal gut bacteria [1,2]. However, some *E. coli* strains carry virulence markers that enable them to cause infections in humans and other animals [3,4]. As such, *E. coli* is included in the ESKAPE-E pathogens (*Enterococcus faecium*, *Staphylococcus aureus*, *Klebsiella pneumoniae*, *Acinetobacter baumannii*, *Pseudomonas aeruginosa*, *Enterobacter spp*., and *E. coli*) considered clinically important [5]. Using these virulence markers, *E. coli* colonize the gut of children and contribute to specific pathophysiology, causing gastroenteritis, invasive infections, urinary tract infections and other diseases [6]. These traits make diarrheagenic *E. coli* (DEC) antigenically distinct

and have been used to classify them into six major pathotypes [7,8]. These pathotypes include Shiga-toxin producing *E. coli* (STEC), enteropathogenic *E. coli* (EPEC), enterotoxigenic *E. coli* (ETEC), enteroaggregative *E. coli* (EAEC), enteroinvasive *E. coli* (EIEC), diffusely adherent *E. coli* (DAEC) and the recent but rare adherent-invasive *E. coli* (AIEC) [9–12].

In low- and middle-income countries (LMICs), like high-income countries (HICs) *E. coli* and rotavirus are among the most important etiological agents of moderate-to-severe diarrhea [13]. Globally, 1.7 billion childhood diarrheal cases are reported annually, resulting in 525,000 deaths [14]. Diarrheagenic *E. coli* causes 80% of diarrheal illnesses in humans in Africa resulting in 70,000 deaths each year with children under the age of five being the most affected [14]. Diarrheal illness is a significant public health concern as it can lead to dehydration and other complications particularly in young children.

Risk factors associated with gut colonization and spread of DEC infection in humans include poor sanitation and hygiene practices such as open defecation, lack of clean water, and inadequate hand washing facilities [15]. Malnutrition is also a significant risk factor for DEC infection as it weakens the immune system and makes individuals more susceptible to infections [16]. Overcrowded living conditions such as in refugee camps and slums can increase the risk of DEC infection due to the ease of person-to-person transmission [17]. Other risk factors include contact with infected individuals and animals, contaminated food and water (including street-vended foods and untreated water sources, such as rivers and lakes) [18]. Individuals with HIV/AIDS are at increased risk of DEC infection due to their weakened immune systems [17,19,20].

As children transition from exclusive breastfeeding at 6-24 months to consumption of complementary foods and water, they are at increased risk of diarrheal disease [21,22]. Recent studies conducted in Kenya on children presenting with diarrhea to various tertiary hospitals have reported DEC prevalence ranging from 34 – 60% [3,23,24]. In Egypt, DEC prevalence among children under five years

of age with diarrhea in the community was 20.6% [11], while a study in Uganda reported a DEC prevalence of 38.2% among children with acute diarrhea [25]. However, there is lack of data on the prevalence of DEC pathotypes among asymptomatic weaning children in the community setting. The aim of this study was to estimate the detection prevalence of DEC pathotypes among asymptomatic children aged 6-24months in a peri-urban community of Nairobi, Kenya, and to identify risk factors for DEC detection.

# Methods

## Ethics consideration

Prior to commencing data collection, ethical approvals were obtained from the Research Ethics Committee of the London School of Hygiene and Tropical Medicine (Ref: 17188) and the Institutional Research Ethics Committee at the International Livestock Research Institute (Ref: ILRI-IREC2019-26). The ILRI-IREC is accredited by the National Commission for Science, Technology, and Innovation in Kenya (NACOSTI). In addition, project and individual student approvals were obtained from NACOSTI (License No: NACOSTI/P/21/10409). All study participants (i.e. adult caregivers) provided written informed consent before entry into the study.

## Study design, population and recruitment strategy

Our cross-sectional study was conducted in Dagoretti Sub-County, Nairobi between 1st May 2021 and 1st October 2021 as part of the Urban Infant Foodscape Project (UIF). Households were stratified and randomly selected based on presence of at least one child 6-24 months old. Dagoretti South sub-county is a peri-urban area covering 29.1km$^2$ and comprises 9.87% (434,208/4,397,073) of Nairobi County's population [26]. It is a low-income peri-urban settlement characterized by high infectious disease burden, inadequate WASH (water, sanitation and hygiene) services and high human

population density [27]. Dagoretti South sub-county was further stratified into the five different administrative areas (wards) and two wards, Uthiru/Ruthimitu and Riruta, were selected based on their proximity to health facilities (Fig 1). A complete list of all active community health volunteers (CHVs) from the two wards was obtained from the sub-county health coordinator. Out of this list, 100 CHVs were randomly selected using random number generator. However, 90 of the 100 CHVs were available to take part in the study. The 90 CHVs provided complete lists of eligible households in their catchment areas that had at least one child 6-24 months old. The households were stratified and up to 7 households were randomly selected from each CHV, resulting in a total of 585 households recruited into the study.

**Fig 1. Map of the study area indicating the wards of Dagoretti South Sub-County within Nairobi County in Kenya**

## Data and Sample collection

A study nurse was paired with a CHV and visited each of the recruited households. After obtaining informed consent from an adult caregiver, the nurse conducted a questionnaire survey. The structured questionnaire covered questions relating to risk factors including poor sanitation and hygienic practices, dietary habits, household size (area and number of people), age and sex of child, contact with infected animals and consumption of contaminated food and water. Water sources were classified based on the WHO/UNICEF Joint Monitoring Program (JMP) “ladder” approach for assessing access to safe water supply, sanitation and hygiene services [28].

After the visit, the nurse left a set of diapers with the caregiver instructing the caregiver to aseptically remove the diaper from the child upon defecation (not touching the inside), wrap and place it into a Ziplock bag labelled with household identification number and date. The diaper was then

collected within 2 hours and transported in cold chain (cool box at 4°C) to the International Livestock Research Institute (ILRI) laboratory for processing within 6 hours of collection.

## Isolation and identification of *E. coli* pathotypes

To isolate *E. coli*, stool samples were directly plated on differential media, MacConkey agar and incubated at 37°C for 18-24h. Up to three lactose fermenting typical colonies appearing red/pink were biochemically tested by stabbing motility-indole-ornithine (MIO) media and streaking a trypticase soy agar (TSA) plate for isolation of pure culture. The TSA plates and MIO tubes were then incubating at 37°C for 18-24h. After incubation, 2-3 drops of Kovacs reagent was added and all motility, indole and ornithine positive colonies considered presumptive *E. coli*. All the presumptive *E. coli* colonies were confirmed by Matrix Assisted Laser Desorption Ionization time of flight, mass spectrophotometry (MALDI-TOF MS), identifying them based on their protein profile.

To identify DEC pathotypes (ETEC, EPEC, EAEC, EIEC, and DAEC), specific virulence markers were used to pathotype the confirmed *E. coli* isolates using multiplex PCR. Typical EPEC was defined by presence of *bfp* (bundle-forming pilus) and *eae* (intimin) and absence of *stx* (Shiga-toxin) while atypical EPEC was characterized by presence of *eae* and absence of *bfp* and *stx* virulence genetic markers. Presence of *elt* (heat-labile toxin-LT) and/or *stI* (heat-stable-toxin-ST) was used to characterize ETEC, while *ipaH* (invasion plasmid antigen H) and *virF* (transcriptional regulator) were used for EIEC screening. Dr-binding adhesin family protein (*daaE*) was characteristic of DAEC while *aafII* (aggregative adherence fimbria II protein AafB) and *pic* (Serine protease autotransporter) were characteristic of EAEC presence [9,12,29]. The PCR conditions and primers are available in S1 File.

For Shiga toxin producing *E. coli* (STEC) seropathotype A (O157:H7) and seropathotype B (non-O157:H7) isolation, stool samples were plated onto Sorbitol MacConkey agar supplemented with

cefixime tellurite (CT-SMAC) and incubated at $37^0$C for 18-24h. Non-sorbitol fermenting colonies (NSFC) transparent or colorless with pale brownish appearance on CT-SMAC are typical of *E. coli* O157:H7. These NSFC were tested for immunochemical reaction with O157:H7 latex reagent (O157 antibody coated latex and control latex) according to manufacturer's instructions [30–32]. Isolates seropositive for O157 and ± H7 flagellar antigen were reported as presumptively positive for *E. coli* O157:H7 since several species cross-react with O157 antiserum. These presumptive colonies were confirmed as *E. coli* using MALDI TOF MS. PCR confirmation of their carriage of virulence markers stx 1 and/or 2 and intimin (eae) gene was done. These were confirmed as enterohemorrhagic *E. coli* (EHEC).

Sorbitol fermenting colonies (SFC) with red or pink appearance on CT-SMAC were biochemically characterized using MIO media as presumptive *E. coli.* These colonies were confirmed as *E. coli* using MALDI TOF MS. These were further pathotyped in multiplex PCR reactions targeting virulence markers specific for each serogroup, to characterize them as non-O157 (non-EHEC) STEC (O26, O45, O103, O111, O121 and O145) [33]. The NSF *Escherichia coli* (ATCC 700728) was used as a positive control, while the sorbitol fermenter *E. coli* (ATCC 25922) was used as a negative control.

## Statistical analysis

Descriptive summary statistics were computed to describe the detection prevalence of the six *E. coli* pathotypes. Presence/absence of an *E. coli* pathotype in stool was the primary outcome. The *lme4* package in R (version 4.2.2) was used to make mixed effects logistic regression models to identify risk factors associated with *E. coli* pathotype(s) carriage in children. Predictor variables considered were household size (number of people residing within a household), livestock ownership, manure/animal feces presence in the compound (as a subset of households keeping livestock), visible sign of rodents, and those relating to food type and food handling practices. Model selection was done in a two-step statistical logistic regression using univariable and backward stepwise multivariable analysis. The CHV identity was used as a random effect to account for clustering in the model regarding risk factors for *E.*

*coli* pathotypes in children. Using a Fisher's test to assess the level of association of predictors identified during univariable analysis, collinearity effect between these predictors was evaluated. Additionally, during the selection process, risk factors that exhibited more than a two-fold change in the logistic regression coefficients were also examined. All predictor variables with p value ≤0.1 during univariable analysis were retained for assessment in the multivariable analysis. A backward stepwise approach was used in the multivariable analysis to select the best model fit. Odds ratios of various predictors of *E. coli* pathotype prevalence and 95% confidence interval were calculated and considered statistically significant if the p-value was < 0.05. To evaluate two-way interactions between predictor variables, a likelihood ratio test was conducted and interactions were deemed significant if the p ≤ 0.05. Generalized Variance Inflation Factors (GVIF) were calculated to check for multicollinearity among the predictor variables. GVIF > 4 were considered a problem and the variable removed from the model. Model validity was checked by comparing the residuals plot to the fitted values plot for each fixed effect to assess for clustering and outliers, and deviations from the empirical and expected quantile distribution were determined to be not significant (p-value > 0.05) using the *simulateResiduals* function from the *DHARMa* package [34]. A Q-Q plot was visualized to detect deviations from the expected distribution including goodness-of-fit tests for correct distribution, overdispersion and outliers.

# Results

## Diarrheagenic *E. coli* prevalence

Stool samples were collected from 540 of the 585 recruited children. The caregivers of the 45 children who did not provide a sample either declined or were unable to do so. Of the 540 samples, 503 (93.1%, 95%CI: 90.8-95.0%) tested positive for *E. coli*. According to the pathotyping results, 113 samples tested positive for diarrheagenic *E. coli*, with the prevalence of DEC in children 6-24 months being 20.9% (95%CI: 17.6 – 24.6%). From the 113 positive samples, 13 samples had co-infections (12 samples positive

for two of the tested DEC pathotypes and one sample positive for three). This translated to a total of 127 isolates of diarrheagenic *E. coli* (Table 1). Enteropathogenic *E. coli* (n= 45, 35.4%) was the most prevalent pathotype (6 isolates tEPEC and 39 aEPEC), followed by EAEC (n=31, 24.4%) and STEC (n=29, 22.8%) among the positive samples.

**Table 1. Prevalence of diarrheagenic *E. coli* isolated from 6-24 months old children in Dagoretti South subcounty, Nairobi**

| ***E. coli* Pathotype** | **No. of children (N=540)** | **Prevalence (95% CI)** |
|---|---|---|
| O157:H7 STEC | 12 | 2.2% (1.3-3.8%) |
| non-O157:H7 STEC | 10 | 1.9% (1.0-3.3%) |
| EPEC | 37 | 6.9% (5.0-9.3%) |
| ETEC | 18 | 3.3% (2.1-5.2%) |
| EAEC | 24 | 4.4% (3.0-6.6%) |
| EIEC | 0 | 0 |
| DAEC | 1 | 0.2% (0.0-1.0%) |
| **Co-infections** | | |
| EPEC/O157:H7/O111 | 1 | 0.2% (0.0-1.0%) |
| EPEC/ETEC | 2 | 0.4% (0.1-1.3%) |
| EPEC/EAEC | 3 | 0.6% (0.2-1.6%) |
| EAEC/ETEC | 1 | 0.2% (0.0-1.0%) |
| EPEC/O157:H7 | 1 | 0.2% (0.0-1.0%) |
| EAEC/O103 | 2 | 0.4% (0.1-1.3%) |

| | | |
|---|---|---|
| EAEC/O157:H7 | 1 | 0.2% (0.0-1.0%) |
| EPEC/O145 | 1 | 0.2% 90.0-1.0%) |
| **Total** | **113** | **20.9% (17.6-24.6%)** |

## Risk factor analysis for Diarrheagenic *E. coli* carriage in children

Table 2 presents the complete univariable analysis results for risk factors associated with DEC carriage in the 6-24m old children. The univariable analysis revealed that the odds of the outcome variable (carriage of DEC) were significantly associated with three predictor variables: age group 12-18m (OR = 1.88, 95%CI: 1.11 – 3.19, p=0.02), consumption of matoke (OR=2.2, 95%CI: 1.40 –3.46, p = 0.001), and consumption of broth (OR=1.90, 95%CI: 1.01– 3.56, p=0.05).

However, some variables lowered the odds of DEC carriage in children. The variables included: diarrhea in the past week (OR=0.76, 95%CI: 0.75 – 0.76, p < 0.001) and food type biscuits (OR=0.21, 95%CI: 0.06 – 0.74, p=0.01) (S1 Table). There was no association between DEC carriage in children and number of people in the household, animal feces on premises, garbage presence on premises, number of households sharing toilets, visible signs of rodents, animal contact and animal ownership (S2 Table).

**Table 2. Results of univariable analysis for risk factors for diarrheagenic *E. coli* carriage in children in peri urban Dagoretti South Sub-County, Nairobi**

| Predictor | Number of observations | Number positive (%) | OR (95%CI) | p-value |
|---|---|---|---|---|
| **Community Health Unit (ward)** | | | | |
| Riruta | 145 | 33 (22.8) | 1 | Reference |
| Ruthimitu | 222 | 42 (34.4) | 0.79 (0.44 – 1.43) | 0.437 |

| | | | | |
|---|---|---|---|---|
| Uthiru | 173 | 38 (22.0) | 0.94 (0.51 – 1.75) | 0.855 |
| **Age groups** | | | | |
| Age group (<12 months) | 170 | 31 (18.2) | 1 | Reference |
| Age group (12-18 months) | 188 | 50 (26.6) | 1.88 (1.11 – 3.19) | **0.02** |
| Age group (>18 months) | 182 | 32 (17.6) | 1.02 (0.58 – 1.80) | 0.94 |
| **Fever in the past one week** | | | | |
| No | 422 | 85 (20.1) | 1 | Reference |
| Yes | 117 | 28 (23.9) | 1.29 (0.77 – 2.16) | 0.33 |
| **Ever breastfed** | | | | |
| No | 7 | 3 (42.9) | 1 | Reference |
| Yes | 533 | 110 (20.6) | 0.32 (0.06 – 1.63) | 0.17 |
| **Type of hand washing facility** | | | | |
| Fixed handwashing facility inside the house | 26 | 5 (19.2) | 1 | Reference |
| Fixed handwashing facility in yard or compound | 52 | 11 (21.2) | 1.19 (0.35 – 4.07) | 0.78 |
| Mobile handwashing object such as a jug/bucket/kettle | 169 | 45 (26.6) | 1.72 (0.58 – 5.15) | 0.33 |
| No handwashing place (in the house, yard or compound) | 293 | 52 (17.7) | 1.01 (0.34 – 2.96) | 0.99 |
| **Main source of drinking water (JMP definition)** | | | | |
| Basic | 475 | 95 (20.0) | 1 | Reference |
| Safe | 65 | 18 (27.7) | 1.55 (0.83 – 2.90) | 0.17 |
| **Use utensils or hands to feed the infant** | | | | |
| Hands | 32 | 4 (12.5) | 1 | Reference |

| | | | | |
|---|---|---|---|---|
| Utensils (e.g. spoon, fork) | 232 | 47 (20.3) | 2.05 (0.65 – 6.44) | 0.22 |
| Hands and utensils | 276 | 62 (22.5) | 2.35 (0.75 – 7.32) | 0.14 |
| **Liquid type consumed (broth)** | | | | |
| No | 457 | 92 (20.1) | 1 | Reference |
| Yes | 83 | 21 (25.3) | 1.43 (0.77 – 2.65) | 0.26 |
| **Food type consumed (pulses/legumes)** | | | | |
| No | 433 | 84 (19.4) | 1 | Reference |
| Yes | 107 | 29 (27.1) | 1.57 (0.94 – 2.64) | 0.09 |
| **Food type consumed (matoke)** | | | | |
| No | 370 | 62 (16.8) | 1 | Reference |
| Yes | 170 | 51 (30.0) | 2.20 (1.40 – 3.46) | **0.001** |
| **Food type consumed (broth)** | | | | |
| No | 470 | 92 (19.6) | 1 | Reference |
| Yes | 70 | 21 (30.0) | 1.90 (1.01 – 3.56) | **0.05** |
| **Food reheating** | | | | |
| Boiled | 114 | 17 (14.9) | 1 | Reference |
| Warmed | 426 | 96 (22.5) | 1.66 (0.92 – 2.98) | 0.09 |
| **Milk reheated** | | | | |
| Boiled | 177 | 29 (16.4) | 1 | Reference |
| Warmed | 363 | 84 (23.1) | 1.49 (0.91 – 2.43) | 0.11 |
| **Neighbor owns chickens** | | | | |
| No | 280 | 53 (18.9) | 1 | Reference |
| Yes | 260 | 60 (23.1) | 1.33 (0.86 – 2.06) | 0.20 |

| | | | | |
|---|---|---|---|---|
| **Neighbor owns cat** | | | | |
| No | 255 | 48 (18.8) | 1 | Reference |
| Yes | 285 | 65 (22.8) | 1.34 (0.86 – 2.10) | 0.20 |
| **Sanitation facility (JMP)** | | | | |
| Unimproved | 73 | 16 | 1 | Reference |
| Limited | 105 | 26 | 1.11 (0.52 – 2.38) | 0.79 |
| Basic | 322 | 57 | 0.73 (0.37 – 1.43) | 0.36 |
| Safely managed | 40 | 14 | 1.84 (0.73 – 4.66) | 0.2 |

## Multivariable analysis

Table 3 summarizes results of the multivariable mixed effect logistic regression model for carriage of diarrheagenic *E. coli* in 6-24months old children. This best fit model had the lowest AIC of 541.0 and included: age group, food type (matoke), food type (pulses/legumes), reheating food (warm enough to eat). The CHV identity was included in the model as a random effect which exhibited an interclass correlation coefficient (ICC) of 0.1. Risk factors associated with DEC carriage were: age group [12-18 months] (OR 1.78; 95%CI 1.03 – 3.07), feeding the infant with “matoke (mashed bananas)” (OR 2.32; 95%CI 1.44-3.73), and feeding infant with pulses/legumes (OR 1.74; 95%CI 1.01 – 2.99). During the stepwise backward variable selection process, all variables were found to have GVIF values below the threshold (GVIF<4), indicating no evidence of multicollinearity among the predictor variables. The final model's conditional R-squared value indicated that the risk factors "feeding the child with matoke", “feeding the child with pulses/legumes”, and "age group of 12-18 months" explained 17.2% of the variation in the data.

**Table 3. Multivariable analysis for carriage of diarrheagenic *E. coli* in children in Dagoretti Sub-County, Nairobi**

| | Pathogenic *E. coli* | | |
|---|---|---|---|
| *Predictors* | *Odds Ratios* | *95%CI* | *p value* |
| Age group [<12 months] | 1 | Reference | Reference |
| Age group [12-18 months] | 1.78 | 1.03 – 3.07 | **0.040** |
| Age group [>18 months] | 1.05 | 0.58 – 1.89 | 0.872 |
| Food type consumed - matoke [Yes] | 2.32 | 1.44 – 3.73 | **0.001** |
| Food type consumed – pulses/legumes [Yes] | 1.74 | 1.01 – 2.99 | **0.046** |
| Food reheated [Warm] | 1.71 | 0.93 – 3.14 | 0.082 |

The final multivariable mixed effects logistic regression model is presented in Fig 2 and the stepwise backward selection followed for model fitting is presented in Table 4. During model validation, no significant clustering patterns of simulated residuals were observed. Further, overdispersion, zero-inflations and outliers tests did not yield any statistically significant results ($p > 0.05$).

**Fig 2. Forest plot showing the final multivariable logistic model for diarrheagenic *E. coli* carriage in children.**

**For each category, the variable, levels, odds ratios (OR) with 95%CI is provided. Horizontal black lines represent 95%CI, while red boxes represent Odds ratios of each variable**.

**Table 4. Comparison of mixed-effects logistic regression models for risk factors for diarrheagenic E. coli carriage in 6-24 months old children.**

| Model | Model formula | AIC |
|---|---|---|
| 1 | pathogenic.e.coli ~ agegroup + surveyFood_Typematoke + surveyFood_Typepulses_legumes + surveyFood_Typebroth + foodreheat1 + (1 \| CHV_ID) | 541.8 |
| 2 | pathogenic.e.coli ~ agegroup + surveyFood_Typematoke + surveyFood_Typepulses_legumes + foodreheat1 + (1 \| CHV_ID) | 541.0 |

For each model, the model formula with their Akaike Information Criterion (AIC) values are provided.

CHV-ID – community health volunteer identity

# Discussion

In this study, we investigate the prevalence of pathogenic *E. coli* among asymptomatic children aged 6-24 months living in a peri-urban community with limited resources and examine any potential risk factors associated with the infection. The results provide insight on the potential food types and food handling practices that are significantly associated with DEC carriage in this vulnerable age group. Our findings of 20.9% prevalence is consistent with reports from clinical isolates in Africa and Asia: 21.2 - 41% in Kenya, 25% in Ethiopia (a review covering 10 articles investigating clinical DEC isolates from under 5 years old), 17-20.6% in Egypt (both healthy adults and children under 5 years of age with diarrhea) and 21% in India with 13 days - 85 year old participants [1,8,11,35–37].

Enteropathogenic *E. coli* was the most prevalent pathotype ~~(35.4%)~~ followed by EAEC ~~(26.5%)~~ and STEC ~~(22.8%)~~. Out of the 45 EPEC isolates, 6 were typical EPEC (positive for both *bfp* and *eae*) and 39 were atypical EPEC (positive only for *eae*). Typical EPEC is more commonly associated with diarrheal disease in infants and young children in developing countries [38]. Since these were presumptive healthy children, this could partly explain the low prevalence of tEPEC (4.6%), which was consistent with the Global Enteric Multicenter Study (GEMS) Kenyan tEPEC results of 3.5 - 5.2% among 0-23 months-old

infants [39]. However, higher prevalence of tEPEC than aEPEC was reported in clinical isolates from Egyptian children [11]. EIEC was not isolated from any sample. Likewise, AIEC was not isolated and it is usually not associated with diarrhea. It is believed to be the etiological agent of Crohn's disease, and so mostly isolated from Crohn's disease patients than from healthy individuals [10]. The same multicenter study (GEMS) reported ST-ETEC prevalence of 7% in 0-23 months old which compares with 9% reported in this study.

Diarrheagenic *E. coli* pathotype co-infections were observed in approximately 11.5% of the samples. This could be due to presence of virulence marker gene combinations like Shiga-toxin genes (*stx1* and 2) and intimin gene (*eae*) seen in STEC and EPEC. These have been used to define hybrid or hetero-pathogenic *E. coli* (like EPEC/STEC Hetero-Pathogen) that causes severe diseases [12]. This example of *E. coli* genomic plasticity was evident in the 2011 German outbreak that involved two pathotypes (EAEC/STEC), leading to recent interest in their significance as more virulent pathogens. Enterohemorrhagic *E. coli* has been linked to global outbreaks, causing severe symptomatology and even fatal outcomes in human hosts [40], therefore its isolation from asymptomatic community children is of both epidemiological and public health concern.

Variables associated with DEC carriage in children at the multivariable model level were: age group 12-18 months, feeding infants with matoke and pulses/legumes. No evidence has been provided linking any food type as a risk factor for DEC pathotype carriage in humans. However, previous studies have indicated that poor personal hygiene practices such as not washing hands before eating or after visiting the toilet, and unhygienic food handling and preparation practices from farm to fork can lead to contamination that eventually impact infant health [18,20,41,42]. Children 12-18 months old are more mobile and curious and they explore their environment by putting things in their mouths and this can increase their exposure to contaminants [43–45]. This stage is also characterized with introduction of more solid foods and declining maternal antibodies [46,47]. At the univariable analysis level, reheating

food and milk to a temperature warm enough to eat was associated with DEC carriage. Optimal culture condition for *E. coli* include incubation at 37°C, thus warming food (and not reheating to boiling) before feeding the infant could be providing an optimal growth condition for DEC. These hygienic practices are likely to be influenced by the household socioeconomic status, therefore recommended intervention strategies should be context specific [18,48–50].

When participants that had diarrhea in the past week were evaluated for DEC positivity, significantly lower odds were observed. This could have been due to fecal shedding that results in lower pathogen positivity as diarrhea subsides and microbiota-mediated pathogen clearance [51]. There was no relationship between DEC carriage and number of household members which has been identified in previous studies as a risk factor together with animal feces/manure within the premises [52,53]. However, there was consistence that livestock ownership had no association with DEC carriage. Further, given the complexity of DEC movement through the different One Health compartments, it is possible that DEC pathotypes move between humans and livestock via polluted environments and/or contaminated foods. This was evident by the significant association of matoke and pulses/legumes with DEC carriage. In a rapidly urbanizing environment like low-resource Dagoretti subcounty in Nairobi, human habitation, livestock keeping and the food supply chain are closely interconnected and indirect rather than direct DEC transmission events should be considered. This phenomenon therefore calls for a sustainable One Health intervention design targeting reduction in zoonotic disease carriage and transmission, and hygienic food handling and preparation practices along the value chain.

## Limitations

Using a PCR-based virulence gene profiling approach for pathotyping *E. coli* offers valuable insights into the pathogenicity potential of the isolates circulating in the community. However, this pathotyping approach has limited gene coverage, passible false negatives, and cannot determine functional expression. Further, with this approach, we were not able to capture emerging virulence

factors thus hindering comprehensive characterization of our *E. coli* isolates. The decision to use this approach was based on cost, excluding more robust whole genome characterization methods, which should be considered for future pathotyping research.

# Conclusion

The results of this study indicate a high prevalence of DEC pathotype carriage in asymptomatic children in the low-resource, densely populated, peri-urban neighborhood of Dagoretti subcounty-Nairobi, that is consistent with the numbers reported in clinical isolates both in Kenya and other countries. There is also evidence of mixed infections that have been shown previously to be indicative of hybrid and hetero-pathotypic infections, implicated in past outbreaks with severe disease symptomatology. This calls for further investigation to elucidate the genomic variation of *E. coli* for better understanding of the epidemiology of these infections. At the multivariable model level, two food types: matoke and pulses/legumes were significantly associated with DEC pathotypes carriage. This may suggest that the handling and preparation practices of these foods could be the cause. Further unidentified risks could be associated with this high carriage of DEC pathotypes in this vulnerable age group. There is no data presently on severe symptoms of gastritis and associated mortality from these asymptomatic DEC pathotype positives. Future research should consider following up asymptomatic community study participants to understand clinical outcomes and risks.

## Conflict of interests

The authors declare that the research was conducted without any commercial or financial relationships that could be perceived as a potential conflict of interest.

## Acknowledgements

The authors wish to thank the participants for their time, the county officials for their support, community health workers and study nurses. Particularly, the authors would like to thank Catherine Mugo, Esther Gichache, Charity Sayo, Nicholas Ireri, John Ng'ang'a, Collins Mbuvi, Jean Amour, Mark Irumva, Immaculate Nyakabi, Hellen Omina and Cynthia Mukanda who helped with sample collection. Special thanks to Michael Ominde of Central Core Unit, ILRI.

## Author contributions

NOO – Conceptualization, investigation, methodology, data curation, formal analysis, writing- original draft

JJNN – Supervision, writing- review and editing

DMM – Formal analysis and writing- review and editing

LO – Investigation and validation

AM –Funding acquisition, supervision, writing- review and editing

CM- Investigation

AK – Investigation

JOW – Investigation

JW - Conceptualization, methodology, writing- review and editing

MAA - Formal analysis

OC – Conceptualization, funding acquisition, methodology, supervision, writing- review and editing

EAJC - Conceptualization, data curation, formal analysis, methodology, supervision, writing- review and editing

## Funding information

The project Development of a comprehensive intervention to address foodborne enteric disease risks among young children living in low-income informal neighbourhoods of Maputo and Nairobi is supported by the Bill & Melinda Gates Foundation (BMGF) and the Foreign, Commonwealth and Development Office (FCDO) of the UK Government (INV-008449) and the study received support from the CGIAR One Health Initiative "Protecting Human Health Through a One Health Approach", which was supported by contributors of the CGIAR Trust Fund (https://www.cgiar.org/funders/).

## Data Availability Statement

Data is contained within the article.

## References

1. Tawfick MM, Elshamy AA, Mohamed KT, El Menofy NG. Gut Commensal Escherichia coli, a High-Risk Reservoir of Transferable Plasmid-Mediated Antimicrobial Resistance Traits. Infect Drug Resist. 2022;15: 1077–1091. doi:10.2147/IDR.S354884

2. Foster-Nyarko E, Pallen MJ. The microbial ecology of Escherichia coli in the vertebrate gut. FEMS Microbiol Rev. 2022;46: 1–22. doi:10.1093/femsre/fuac008

3. ~~Rono SJ, Kakai PR, Esamai PF. Pathotypes and virulence markers in Escherchia Coli associated with diarrhoea among HIV seropositive and seronegative children below five. Eur Sci J. 2014;10: 180–195.~~

4. Pakbin B, Brück WM, Rossen JWA. Virulence factors of enteric pathogenic Escherichia coli: A review. ~~Int J Mol Sci. 2021;22~~. doi:10.3390/ijms22189922

5. Murray CJ, Ikuta KS, Sharara F, Swetschinski L, Robles Aguilar G, Gray A, et al. Global burden of bacterial antimicrobial resistance in 2019: a systematic analysis. Lancet. 2022;399: 629–655. doi:10.1016/S0140-6736(21)02724-0

6. Ducarmon QR, Zwittink RD, Willems RPJ, Verhoeven A, Nooij S, van der Klis FRM, et al. Gut colonisation by extended-spectrum β-lactamase-producing Escherichia coli and its association with the gut microbiome and metabolome in Dutch adults: a matched case-control study. ~~The Lancet Microbe. 2022;5247~~: 1–9. doi:10.1016/s2666-5247(22)00037-4

7. Bolton DJ, O'Sullivan J, Duffy G, Baylis CL, Tozzoli R, Wasteson Y LS. Methods for detection and molecular characterisation of pathogenic Escherichia col. Ashtown Food Research Centre, Ashtown, Ireland; 2007.

8. Nyanga PL, Onyuka J, Webale MK, Were T, Budambula V. Escherichia coli pathotypes and Shigella sero-groups in diarrheic children in Nairobi city, Kenya. ~~Gastroenterol Hepatol from Bed to~~

Bench. 2017;10: 220–228.

9. Chandra M, Cheng P, Rondeau G, Porwollik S, McClelland M. A single step multiplex PCR for identification of six diarrheagenic E. coli pathotypes and Salmonella. Int J Med Microbiol. 2013;303: 210–216. doi:10.1016/j.ijmm.2013.02.013

10. Robins-Browne RM, Holt KE, Ingle DJ, Hocking DM, Yang J, Tauschek M. Are Escherichia coli pathotypes still relevant in the era of whole-genome sequencing? Front Cell Infect Microbiol. 2016;6: 1–9. doi:10.3389/fcimb.2016.00141

11. Khairy RMM, Fathy ZA, Mahrous DM, Mohamed ES, Abdelrahim SS. Prevalence, phylogeny, and antimicrobial resistance of Escherichia coli pathotypes isolated from children less than 5 years old with community acquired- diarrhea in Upper Egypt. BMC Infect Dis. 2020;20: 1–9. doi:10.1186/s12879-020-05664-6

12. Santos AC de M, Santos FF, Silva RM, Gomes TAT. Diversity of Hybrid- and Hetero-Pathogenic Escherichia coli and Their Potential Implication in More Severe Diseases. Front Cell Infect Microbiol. 2020;10: 1–11. doi:10.3389/fcimb.2020.00339

13. Kotloff KL, Blackwelder WC, Nasrin D, Nataro JP, Farag TH, Van Eijk A, et al. The Global Enteric Multicenter Study (GEMS) of diarrheal disease in infants and young children in developing countries: Epidemiologic and clinical methods of the case/control study. Clin Infect Dis. 2012;55. doi:10.1093/cid/cis753

14. WHO (World Health Organization). Diarrhoeal disease WHO. 2017; 3. Available from: https://www.who.int/news-room/fact-sheets/detail/diarrhoeal-disease;

15. Barnes AN, Anderson JD, Mumma J, Mahmud ZH, Cumming O. The association between domestic animal presence and ownership and household drinking water contamination among peri-urban communities of Kisumu, Kenya. PLoS One. 2018;13: 1–17. doi:10.1371/journal.pone.0197587

16. Macallan D. Infection and malnutrition. Medicine (Baltimore). 2009;37: 525–528. doi:10.1016/j.mpmed.2009.07.005

17. Mekonnen M, Geda B, Teklemariam Z, Weldegebreal F, Balakrishnan S. Prevalence of childhood diarrhea and associated risk factors in Dire Dawa, eastern Ethiopia. J Public Heal. 2018;26: 29–37. doi:10.1007/s10389-017-0843-y

18. Hoffmann V, Simiyu S, Sewell DK, Tsai K, Cumming O, Mumma J, et al. Milk Product Safety and Household Food Hygiene Influence Bacterial Contamination of Infant Food in Peri-Urban Kenya. Front Public Heal. 2022;9: 1–12. doi:10.3389/fpubh.2021.772892

19. Gautam OP, Schmidt WP, Cairncross S, Cavill S, Curtis V. Trial of a novel intervention to improve multiple food hygiene behaviors in Nepal. Am J Trop Med Hyg. 2017;96: 1415–1426. doi:10.4269/ajtmh.16-0526

20. Gautam OP, Curtis V. Food hygiene practices of rural women and microbial risk for children: Formative research in Nepal. Am J Trop Med Hyg. 2021;105: 1383–1395. doi:10.4269/ajtmh.20-0574

21. Hörnell A, Lagström H, Lande B, Thorsdottir I. Breastfeeding, introduction of other foods and effects on health: a systematic literature review for the 5th Nordic Nutrition Recommendations. Food Nutr Res. 2013;57: 20823. doi:10.3402/fnr.v57i0.20823

22. Bagamian KH, Anderson JD, Muhib F, Cumming O, Laytner LA, Wierzba TF, et al. Heterogeneity in enterotoxigenic Escherichia coli and shigella infections in children under 5 years of age from 11 African countries: a subnational approach quantifying risk, mortality, morbidity, and stunting. Lancet Glob Heal. 2020;8: e101–e112. doi:10.1016/S2214-109X(19)30456-5

23. Iijima Y, Oundo JO, Hibino T, Saidi SM, Hinenoya A, Osawa K, et al. High Prevalence of Diarrheagenic Escherichia coli among Children with Diarrhea in Kenya. Jpn J Infect Dis. 2017;70: 80–83. doi:10.7883/yoken.JJID.2016.064

24. Webale MK, Wanjala C, Guyah B, Shaviya N, Munyekenye G, Nyanga PL, et al. Epidemiological patterns and antimicrobial resistance of bacterial diarrhea among children in Nairobi city, Kenya. Gastroenterol Hepatol - From Bed to Bench. 2020;13.

25. Masiga F, Kigozi E, Najjuka CF, Kajumbula H, Kateete DP. Diarrhoeagenic Escherichia coli isolated from children with acute diarrhoea at Rakai hospital, Southern Uganda. Afr Health Sci. 2022;22: 581–588. doi:10.4314/ahs.v22i1.67

26. KNBS. Kenya population and housing census volume 1: Population by County and sub-County. Kenya National Bureau of Statistics. 2019.

27. Dominguez-Salas P, Alarcón P, Häsler B, Dohoo IR, Colverson K, Kimani-Murage EW, et al. Nutritional characterisation of low-income households of Nairobi: socioeconomic, livestock and gender considerations and predictors of malnutrition from a cross-sectional survey. BMC Nutr. 2016;2: 1–20. doi:10.1186/s40795-016-0086-2

28. WHO/UNICEF. Joint Monitoring Programme for Water Supply , Sanitation and Hygiene. Guidance note to facilitate country consultation on JMP estimates for drinking water. 2021; 1–7.

29. Hegde A, Ballal M, Shenoy S. Detection of diarrheagenic Escherichia coli by multiplex PCR. Indian J Med Microbiol. 2012;30: 279–284. doi:10.4103/0255-0857.99485

30. Bialka KL, Demirci A. Decontamination of Escherichia coli O157:H7 and Salmonella enterica on blueberries using ozone and pulsed UV-light. J Food Sci. 2007;72: 391–396. doi:10.1111/j.1750-3841.2007.00517.x

31. Hamzah AM, Hussein AM, Khalef JM. Isolation of Escherichia coli 0157 : H7 Strain from Fecal Samples of Zoo Animal. 2013;2013. doi:10.1155/2013/843968

32. Riley DG, Gray JT, Loneragan GH, Barling KS, Chase CC. Escherichia coli O157:H7 Prevalence in Fecal Samples of Cattle from a Southeastern Beef Cow-Calf Herd. J Food Prot. 2003;66: 1778–1782. doi:10.4315/0362-028X-66.10.1778

33. Paddock Z, Shi X, Bai J, Nagaraja TG. Applicability of a multiplex PCR to detect O26, O45, O103, O111, O121, O145, and O157 serogroups of Escherichia coli in cattle feces ~~1~~. Vet Microbiol. 2012;156: 381–388. doi:10.1016/j.vetmic.2011.11.017

34. Hartig F. DHARMa: Residual Diagnostics for Hierarchical (Multi-Level / Mixed) Regression Models. R package version 0.2.0. 2018. p. https://CRAN.R-project.org/package=DHARMa.

35. Zenebe T, Mitiku M, Alem Y. Prevalence of Escherichia coli in Under-Five Children with Diarrhea in Ethiopia: A Systematic Review and Meta-Analysis. Int J Microbiol. 2020;2020. doi:10.1155/2020/8844294

36. Thakur N, Jain S, Changotra H, Shrivastava R, Kumar Y, Grover N, et al. Molecular characterization of diarrheagenic Escherichia coli pathotypes: Association of virulent genes, serogroups, and antibiotic resistance among moderate-to-severe diarrhea patients. J Clin Lab Anal. 2018;32. doi:10.1002/jcla.22388

37. Shah M, Odoyo E, Wandera E, Kathiiko C, Bundi M, Miringu G, et al. Burden of rotavirus and enteric bacterial pathogens among children under 5 years of age hospitalized with diarrhea in suburban and rural areas in Kenya. Jpn J Infect Dis. 2017;70: 442–447. doi:10.7883/yoken.JJID.2016.398

38. Trabulsi LR, Keller R, Tardelli Gomes TA. Typical and atypical enteropathogenic Escherichia coli. Emerg Infect Dis. 2002;8: 508–513. doi:10.3201/eid0805.010385

39. Kotloff KL, Nataro JP, Blackwelder WC, Nasrin D, Farag TH, Panchalingam S, et al. Burden and aetiology of diarrhoeal disease in infants and young children in developing countries (the Global Enteric Multicenter Study, GEMS): A prospective, case-control study. Lancet. 2013;382: 209–222. doi:10.1016/S0140-6736(13)60844-2

40. Gomes TAT, Elias WP, Scaletsky ICA, Guth BEC, Rodrigues JF, Piazza RMF, et al. Diarrheagenic Escherichia coli. Brazilian J Microbiol. 2016;47: 3–30. doi:10.1016/j.bjm.2016.10.015

41. Berendes D, Knee J, Sumner T, Capone D, Lai A, Wood A, et al. Gut carriage of antimicrobial resistance genes among young children in urban Maputo, Mozambique: Associations with enteric pathogen carriage and environmental risk factors. PLoS One. 2019;14: e0225464. doi:10.1371/journal.pone.0225464

42. Odeyemi OA. Public health implications of microbial food safety and foodborne diseases in developing countries. Food Nutr Res. 2016;60: 20–22. doi:10.3402/fnr.v60.29819

43. Ferguson A, Solo-Gabriele H. Children’s exposure to environmental contaminants: An editorial reflection of articles in the ijerph special issue entitled, “children’s exposure to environmental contaminants.” ~~Int J Environ Res Public Health. 2016;13~~. doi:10.3390/ijerph13111117

44. Gorman Ng M, Davis A, Van Tongeren M, Cowie H, Semple S. Inadvertent ingestion exposure: Hand-and object-to-mouth behavior among workers. J Expo Sci Environ Epidemiol. 2016;26: 9–16. doi:10.1038/jes.2014.71

45. Xue J, Zartarian V, Moya J, Freeman N, Beamer P, Black K, et al. A meta-analysis of children’s hand-to-mouth frequency data for estimating nondietary ingestion exposure. Risk Anal. 2007;27: 411–420. doi:10.1111/j.1539-6924.2007.00893.x

46. Checkley W, Buckley G, Gilman RH, Assis AM, Guerrant RL, Morris SS, et al. Multi-country analysis of the effects of diarrhoea on childhood stunting. Int J Epidemiol. 2008;37: 816–830. doi:10.1093/ije/dyn099

47. Chatziprodromidou IP, Chatziantoniou S, Vantarakis G, Vantarakis A. Risk Factor Analysis of Children’s Exposure to Microbial Pathogens in Playgrounds. Risk Anal. 2022;42: 334–343. doi:10.1111/risa.13752

48. Simiyu S, Czerniewska A, Aseyo ER, Baker KK, Cumming O, Mumma JAO, et al. Designing a food hygiene intervention in low-income, peri-urban context of Kisumu, Kenya: Application of the trials of improved practices methodology. Am J Trop Med Hyg. 2020;102: 1116–1123. doi:10.4269/AJTMH.19-0629

49. Awiti J, Mumma O, Cumming O, Simiyu S, Czerniewska A, Aseyo RE, et al. Infant Food Hygiene and Childcare Practices in Context : Findings from an Urban Informal Settlement in Kenya. 2020;102: 220–222. doi:10.4269/ajtmh.19-0279

50. Simiyu S, Aseyo E, Anderson J, Cumming O, Baker KK, Dreibelbis R, et al. A Mixed Methods Process Evaluation of a Food Hygiene Intervention in Low-Income Informal Neighbourhoods of Kisumu, Kenya. Matern Child Health J. 2022; 824–836. doi:10.1007/s10995-022-03548-6

51. Endt K, Stecher B, Chaffron S, Slack E, Tchitchek N, Benecke A, et al. The microbiota mediates pathogen clearance from the gut lumen after non-typhoidal salmonella diarrhea. PLoS Pathog. 2010;6. doi:10.1371/journal.ppat.1001097

52. Muloi D, Kiiru J, Ward MJ, Hassell JM, Bettridge JM, Robinson TP, et al. Epidemiology of antimicrobial-resistant Escherichia coli carriage in sympatric humans and livestock in a rapidly urbanizing city. Int J Antimicrob Agents. 2019;54: 531–537. doi:10.1016/j.ijantimicag.2019.08.014

53. Muloi DM, Hassell JM, Wee BA, Ward MJ, Bettridge JM, Kivali V, et al. Genomic epidemiology of Escherichia coli: antimicrobial resistance through a One Health lens in sympatric humans, livestock and peri-domestic wildlife in Nairobi, Kenya. BMC Med. 2022;20: 1–11. doi:10.1186/s12916-022-02677-7

# Supporting information

S1 File. PCR conditions for Escherichia coli pathotyping

S1 Table. Variables lowering Odds of DEC carriage in children

S2 Table. Variables with no association with DEC carriage in children

Figure

Click here to access/download;Figure;Fig 2. Forest plot for multivariable model.tif

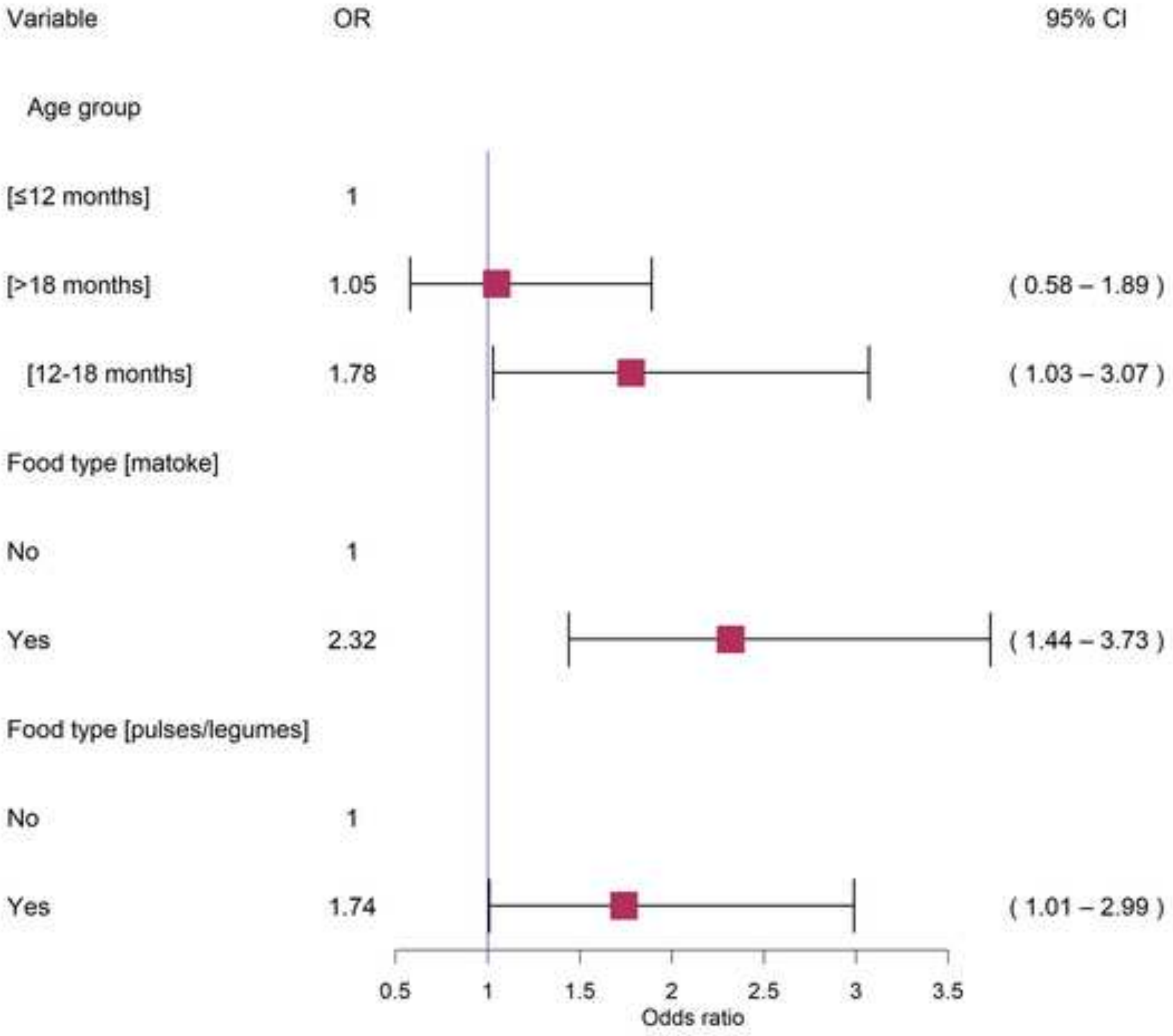

Figure

Click here to access/download;Figure;Fig 1. Map of study area.tif

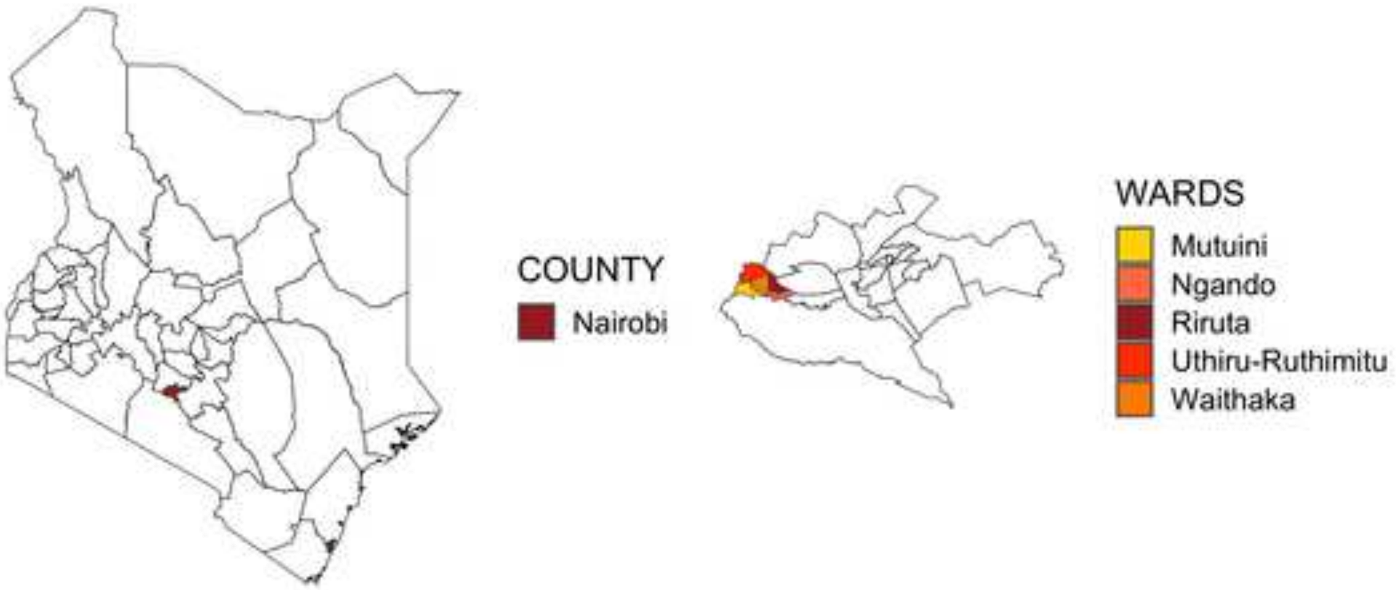

Click here to access/download
**Supporting Information**
S1 File.docx

Click here to access/download
**Supporting Information**
S1 Table.docx

Click here to access/download
**Supporting Information**
S2 Table.docx
