# Supplementary material for: Risk factors for diarrheagenic Escherichia coli infection in children aged 6–24 months in peri-urban community, Nairobi, Kenya
Source: PLOS Glob Public Health. 2023 Nov 22;3(11):e0002594. doi: 10.1371/journal.pgph.0002594 (PMC10664883; doi:10.1371/journal.pgph.0002594)
Supplement: Attachment — Submitted filename: Response to Reviewers.docx [file pgph.0002594.s007.docx]

Rebuttal

**To the Editor**

The reference list is now complete.

1. The following have been updated:
i. Journal abbreviations
ii. Year of publication including month and day
iii. No retracted paper has been cited.
2. Copyright issues
i. Fig 1. Source of the shapefiles has been provided in the revised manuscript (lines 113-114)
ii. Fig 2. was generated using R.
3. Figures using PACE: Fig 1, 2 and 3 were uploaded to PACE and uploaded as TIF files

**Reviewer #1**

1. **Several potential risk factors were thought to contribute to infection, was climate associated conditions also considered eg. an increase in infection in the rainy season compared to the dry season?**

Response: *climate associated conditions were not investigated in this study since this was a cross-sectional study. Moreover, the data for this study was collected during the dry season from May- October*

2. **Are environmental samples planned to be taken?**

Response: *We collected samples from livestock but this will be reported separately.*

3. **Are there any interventions that are in place regarding safe food preparation practices in the county?**

Response: *Interventions are part of a phase 2 of the Urban Infant Foodscape (UIF) project, which will be designed using results stated in this submission.*

4. **A follow-up study as to how of the children infected with *E. coli* actually went on to develop diarrhoeal disease, would be interesting as well.**

Response: *We agree this would be interesting. However, it was/is not within the scope of the current project design.*

**Reviewer #2**

1. **Comment: Though it's not the objective of the study it would have been great if the study was a cohort study and follow the child whether they develop diarrhea or not and which patient depending on the pathotype will have complications.**

Response: *We agree a longitudinal study is ideal. And we are exploring if this could be included in future projects.*

**Reviewer #3**

1. **On page 5- would suggest explaining removal of diaper 'aseptically'- as this surely is not possible in the community.**

*Response: We deleted the word aseptically (line 124 in the revised manuscript). The nurse explained and demonstrated to the caregiver how to put on the diaper, remove it from the child and wrap it without touching the inside contents.*

2. **In results section on page 5, could you be more specific about why caregivers were 'unable to do so' (provide a sample)- was this because the child was unwell or had loose stools?**

*Response: these children did not produce a stool within the stipulated time- either failed to produce stool for the entire 3 days (allowed for follow-up) or produced stool at a time the validity of the sample could not be guaranteed (e.g. late evening or at night).*

3. **Does Table 1 have p-values?**

Response: *No. This table indicates the prevalence with 95% CI values for each pathotype. This is not a comparison and hence no need for P values.*

4. **Should there be a demographics table before Table 1- which describes the sampled populations?**

Response*: a paragraph has been added briefly describing the age and gender of the sampled children (lines 197-203). A variable (Infant gender) has been added into table 2 of the univariable analysis.*

5. **Apologies I find Table 4 difficult to interpret- if just formulas for the regression model, perhaps more appropriate for an Appendix section.**

Response: *Table 4 describes the stepwise backward selection of the multivariable model. We have moved it to the Supplementary material (S3 Table). Description is given in lines 248-249 in the revised manuscript.*

**Reviewer #4**

1. Line 29: Comment: **Please include "asymptomatic healthy children" as it´s not mentioned in the abstract**

Response: Line 20 of the revised manuscript the word “presumptive” has been replaced with “*asymptomatic*.”

2. Line 44: comment: **Please include the importance of ESKAPE-E group, with particular focus on AMR**

Response: *ESKAPE-E pathogens are clinically important. The clinical importance of these pathogens include antimicrobial resistance. We have added a sentence here to capture this.*

3. Line 66,67: Comment: **Merge phrase with "malnutrition", line 60**

Response: Has been merged with line 65 in the revised manuscript to avoid repetition. It now reads *“Malnutrition and HIV/AIDS infections are also a significant risk…..”*

4. Line 88 in the pdf: Comment: **Readers might be unfamiliar with this project. Please reference and/or briefly explain its key characteristics**

Response: lines 93-97 added to give a brief description of the “UIF project.” The added description reads *“The UIF project aimed to estimate foodborne disease (FBD) burden in young children in a low-income, high density urban environment by quantifying microbial contamination of pediatric foods and evaluating the risk factors for contamination and exposure across three domains: household, market and the production/supply chain and design intervention.”*

5. Line 94 in the pdf: Comment: **Table 2 uses Uthiru and Ruthimitu as different wards, totaling 3 community health units. Please explain and provide consistent numbers.**

Response: *Uthiru/Ruthimitu make one administrative area (ward) while they represent different community health units (the reason for having them separate in table 2). The word “ward” has been removed from table 2 to avoid the confusion.*

6. Line 103 on pdf: Comment: **Maps is extremely helpful to contextualize the study settings. Additionally, I'd suggest the authors to:**

**1. Ensure a high resolution figure is used in the final version of the manuscript**

**2. Please put the country map in context with the African continent**

Response:

i. The figure was developed using PACE as suggested to improve resolution.
ii. We have added an inset of the African continent indicating Kenya
iii. Source of shapefiles for Fig 1 has been provided in the Figure legend (lines 113 and 114). Added to the legend: *“Base layers were provided by the Humanitarian Data Exchange (https://data.humdata.org/) and ArcGIS Hub https://hub.arcgis.com/datasets”*
7. Line 119 in the pdf: Comment: **Isolation and identification of E. coli were adequately described in details. However, considering the complexity of the process and nomenclature involved, a figure/flowchart would reduce text length and provide a simpler and better panorama of this significant section of the study**

Response: *We appreciate the suggestion to include a flowchart and we have provided this. However, we kept the text to explain the isolation and identification of E. coli as this best describes this section in adequate detail.*

8. Lines 145,146. Comment: **Molecular evaluation of virulence markers were cited in the identification process but the results are not presented in the manuscript. Please provide data (if available) or remove/rewrite phrase.**

Response: *Further details on how this identification was done are provided in S1 File. The pathotyping results provided in table 1 are based on this molecular identification using PCR*.

9. **Line 181 in the pdf: I have included the % (i.e. 92.3%).**
10. Line 188: comment: **tEPEC and aEPEC were not used previously, please provide full term description (lines 47-50)**

Response: full description of tEPEC and aEPEC has been inserted in lines 52-55 of the revised manuscript. It now reads: *“Enteropathogenic E. coli can be typical (carrying both bfp and eae genes) or atypical (carrying only eae gene)….”*

11. **Line 191 in the pdf** : the word “*asymptomatic*” has been inserted in line 215 of the revised manuscript.
12. Table 1: Comment: **Calculation results in n=114, please review calculation**

Response: *Totals in table 1 has been reviewed and there were some errors in the tabulations. We have checked these. The total remains 113 with prevalence of 20.9% (95%CI 17.8-24.5). Corrected lines include lines 209-213 in the revised manuscript.*

13. Line 205 in the pdf: the word “*asymptomatic*” has been inserted into line 230 of the revised manuscript
14. Table 2: Comment: **The reason Riruta was chosen as reference was not cited until now (lines 93-95). Please provide a brief explanation.**

Response: *There was not a specific reason for choosing Riruta as the reference. The R package assigns this to the level that comes first in the alphabet. Since there was no significant association with community health unit we did not see a reason to manipulate this.*

15. Line 214 of the pdf: comment: **Matoke was cited earlier in the manuscript but never explained until now. Please move the description to the first appearance of this term.**

Response: “matoke” has now been first defined in line 28 of the revised manuscript to read *“matoke (mashed bananas)”. We have kept the definition throughout at all mentions for readers who are not familiar with this term.*

16. Line 221 of the pdf: the word “*asymptomatic”* has been inserted to line 245 of the revised manuscript.
17. Line 247 of the pdf: comment: **These are raw results and, therefore, should not be presented in Discussion. Please remove, move to Results or rewrite as discussion. Finally, the terms typical and atypical EPEC are used in their full length only here. Please move these definitions to the first time these terms are cited in the text (lines 47-50)**

Response: *Definition of typical and atypical EPEC removed since these have now been defined in lines 52-53 of the revised manuscript where they first appear. The raw data in lines 270-272 have been deleted.*

18. Line 254 of the pdf: comment: **Probably "trigger" would fit better in this assumption**

Response: *the word “agent” has been replaced by the word “trigger” in line 278 of the revised manuscript.*

19. Line 270 of the pdf: Comment: **Since no evidence has been provided linking any food type as a risk factor for DEC pathotype carriage in humans, this topic deserves a more robust discussion. For readers not aware of selected practices and dietary habits in the Africa: is there any peculiarity in matoke, pulses/legumes or their preparation that could be linked to the increased risk of carriage? Any peculiarity the readers should know to better understand the dietary habits of the study population?**

Response: I have inserted lines 292-297 (in the revised manuscript) to help the reader understand the dietary habits of the study area. The inserted lines read: “*Grains and mashed bananas represent common staples in the Kenyan diet, while pulses/legumes and matoke were the predominant food categories investigated in this study. These foods are easily obtained from street vendors, and their use in households may be attributed to affordability, accessibility, nutritional composition, ease of preparation, and serving, making them popular choices for pediatric nutrition within the peri-urban settlement. Although…”*

20. Line 286 in the pdf: Comment: **Provide more discussion on this potential association. Some useful references may include: https://pubmed.ncbi.nlm.nih.gov/29164137/**

Response: I inserted lines 315 and 316 in the revised manuscript. It now reads *“Livestock keeping is a common practice in this rapidly urbanizing environment, with interaction being possible even with non-livestock keeping households [29,54,55]”. Some references have been added.*

21. Line 352 in the pdf: Comment: **References must be formatted using reference management software. I've selected some to be reviewed but virtually all require to be re-formatted.**

Response: The entire reference list has been reformatted using EndNote (Vancouver); *Reference 55* added to line 316 of the revised manuscript.
